# Report on Progress of Trench Fever Investigations1.Read by Major R. P. Strong on March 15, 1918, at the Pasteur Institute, Paris, at the meeting of the Medical Research Committee of the American Red Cross. A similar report of the scientific results was presented at the meeting of the Inter-Allied Sanitary Conference, Paris, March 13, 1918, and is preserved in the archives of that commission.

**Published:** 1918-03

**Authors:** 


### REPORT ON PROGRESS OF TRENCH FEVER INVESTIGATIONS[1]

OF

TRENCH FEVER COMMISSION OF MEDICAL RESEARCH COMMITTEE
AMERICAN RED CROSS

Consisting of

Majors R. P. STRONG, Homer F. SWIFT, and E. L. OPIE;
Captains Ward J. MACNEAL, Walter BAETJER,
and A. M. PAPPENHEIMER, M. O. R. C.;
and Lieutenant A. D. PEACOCK, R. A. M. C. (T.)

At the first meeting of the Medical Research Committee of the A. R. C., held in October, 1917, and presided over by Major Alexander Lambert, Chief Surgeon, American Red Cross, one of us[2] suggested the subject of the transmission of trench fever as being one of the most important for investigation in connection with the loss of man-power in some of the armies on the western front, and as being a more urgent problem for study in connection with the prevention of this disease than that of its etiology, the latter problem having already been studied extensively. It was requested that a statement be submitted at the next meeting of the committee regarding our present knowledge of this disease. This report was submitted at the meeting of the Research Committee held in November, and was published in the *Medical Bulletin* of the A. R. C. for December. Following the reading of this report it was voted that the subject of the method of transmission of the disease and an investigation regarding the infectious properties of the blood in trench fever, were important problems for investigation, and that

1. Read by Major R. P. Strong on March 15, 1918, at the Pasteur Institute, Paris, at the meeting of the Medical Research Committee of the American Red Cross. A similar report of the scientific results was presented at the meeting of the Inter-Allied Sanitary Conference, Paris, March 13, 1918, and is preserved in the archives of that commission.

2. Major Strong.

such investigations should be undertaken. A trench fever committee, consisting of Majors Cushing, Swift, and Strong, was therefore appointed. At this and at subsequent meetings one of us [1] was asked to plan and take charge of the work, select the personnel, for the same, and begin the experiments as soon as possible.

Major Cushing was asked (in November) to confer with the British authorities in reference to cooperation upon these investigations, and to see if either the services of Mr. Bacot, of the Lister Institute, or of Lieut. A. D. Peacock, R. A. M. C., could be secured to help in the work[2]. He received considerable encouragement from Col. T. R. Elliot, R. A. M. C., and General Sir Wilmot Herringham later wrote inviting one of us to attend a meeting of the Medical Investigation Committee of the B. E. F., held December 8th, 1917. There were present at this meeting Colonel Sir William Leishman, Chairman, Major General Sir John Rose Bradford, Major General Sir Wilmot Herringham, and Colonel W. O. Beveridge. This committee then voted to accept our offer of assistance in the study of trench fever and after some discussion agreed to turn over to us for investigation two subjects; first, the method of transmission of the disease, and, second, the question of the infectious properties of the blood, with a view to repeating and confirming if possible the previous work of Major McNee, R. A. M. C.

It was finally arranged, on the recommendation of General Sir William Macpherson A. D. G. M. S., by Field Marshal Sir Douglas Haig, the Commander-in-Chief of the British Armies in France, and General Pershing, the Commanding General of the American Expeditionary Forces, that this work should be carried on at a stationary hospital of the B. E. F. which was near enough to the line, where cases of trench fever in the very early stages of the disease could be secured. Perhaps the most important problem was the securing of volunteers for the experiments, for, since the disease could not be transmitted to animals, it was clear that unless volunteers were secured, the experiments could not be performed. Colonel M. W. Ireland, U. S. M. C., successfully undertook this task, and the Commander-in-Chief of our army on December 22nd gave permission to General Bradley, our Chief Surgeon, to have these experiments made on volunteers from the American army provided, as Colonel Ireland had outlined, that each volunteer was to have it carefully explained to him what he was volunteering for, and that after this explanation he should sign a paper that he was willing to undergo the experiment.

1. Major Strong.

2. The Services of Lieut. Peacock were secured, Jan. 26, 1918.

In the meantime the necessary equipment with which to carry on the work had been selected and gathered together in Paris, the funds for the purchase of the same being supplied by the A. R. C. through the instructions of Major Lambert, its Chief Surgeon. The laboratory apparatus and supplies, tents, lumber for latrines, ablution chambers, flooring, drainage and water pipes, window glass, etc., were shortly afterwards received at the stationary hospital where the experiments were to be carried on. Sixty volunteers from the U. S. Army arrived a few days later and 8 others subsequently.

The first week was occupied largely in the examination of the volunteers and in converting the empty hut supplied us into a laboratory, in erecting tents, and in building latrines, ablution chambers, construction of drains, etc., in which work we were greatly assisted by the British personnel of the hospital. Clinical histories of the volunteers were taken, physical examination made, and bacteriological and serological examinations of the blood, urine, and faeces performed, in order that complete records of the men might be available before the experiments were made. All the men used in the experiments were healthy and robust.

Four-hourly temperature charts were kept of all of them from the date of their arrival. A plan for semi-weekly inspection and bath, and for complete sterilization of clothing once a week, was arranged, and other precautions were taken to prevent the men from becoming lousy by natural means during their station at this Hospital. In order that the results obtained in the experiments should be unquestioned, the men of the detachment also were segregated from the B. E. F. hospital personnel and patients.

The experiments were conducted upon the plan already described at a previous meeting, relating to the subjects of the transmission of the disease and of the determination as to whether the organism causing it was situated in the blood, and particularly in the blood cells, as had been previously reported.

Thirty-four men have been employed in the blood inoculation experiments, and these have been inoculated either with blood or some constituent element of it taken from trench fever cases in the febrile stage of the disease. Of these, twenty three have developed typical trench fever. Sixteen of these were inoculated with whole blood, of which number 15 have developed the disease. The number of individuals inoculated with whole blood may seem large, but it should be observed in this connection that in every series of the experiments it was necessary to determine at the time the presence of the virulent organism in the trench fever blood employed. Unless its presence could be demonstrated in the whole blood used at the time and under the conditions of the other experiments, no conclusions could be drawn regarding its apparent absence in any part of the blood or in filtrates of it.

Five individuals were inoculated with clear unfiltered plasma of the blood, free from cells, all of whom have contracted the disease; four were inoculated with washed corpuscles, of whom three have developed trench fever; five with plasma or serum filtered through Berkefeld filters, of whom none has contracted the disease; and two with extracts of ground corpuscles, filtered through Berkefeld filters, of whom none has contracted the disease. From these experiments it is evident that the organism causing the disease is present in the blood and particularly in the plasma of the blood. It is also evident that it may be centrifuged down with the corpuscles of the blood and is not washed free from them by saline solution, but sometimes appears to adhere to them in sufficient quantity to produce the disease after injection of the corpuscles. The organism is perhaps also brought down by the fibrin meshwork of the clot when the clotted blood is centrifuged. From the experiments performed up to this time it would appear that the organism is either not present in the filtrates or at least is not present in sufficient amount, or in proper condition, to produce the disease following inoculation. It may remain behind or in the filter, in which case it is probably of sufficient size to be visible under the microscope under certain conditions or during some stage of its life history.

The incubation period of the disease produced by inoculation with blood varies from 5 to 20 days. Of 20 cases inoculated with whole blood or plasma : in 9 cases it was 5 days; in 4 cases 6 days; in 2 cases 7 days; in 1 case 9 days; in 2 cases 13 days; in 1 case 19 days and 1 case 20 days. In those cases, with one exception, in which the incubation period was long (2 cases of 13 days and 1 of 20 days) the individuals were inoculated with blood either taken late in the disease (6th or 7th day) or during the relapse of the fever. The blood appears from the inoculation experiments to be most infectious on the 1st and 2nd days of the disease, and more infectious during the first attack than during the relapse. In the first series of inoculations the blood was taken from 7 cases of trench fever occurring naturally in members of the B. E. F. and was injected from each case into a single American volunteer. All of the seven volunteers developed the disease. The organism causing the disease has in addition been carried from three trench fever cases through three more generations in man from the original case in three series of experiments.

In the inoculation experiments varying susceptibility to the disease and relative immunity of some individuals are demonstrated by the length of the incubation period, the severity of the attack, and even, in one instance, by failure, so far, to develop trench fever. With reference to these instances of relative immunity by parallel experiments it was shown that the organism was at the time alive and sufficiently virulent in the blood to infect other individuals in a shorter time, while the more immune individuals inoculated at the same time developed the disease later, or, in one instance, has remained healthy.

In relation to transmission of the disease by the louse *Pediculus corporis* 26 men have been subjected to experiment. Of these, 22 have harbored lice which have bitten trench fever cases in the febrile stages of the disease, and the remainder have harbored, for the same period of time, normal, uninfected lice which have not bitten trench fever cases. Eight other volunteers living under exactly the same conditions as those contracting trench fever and kept free from lice, as well as the 4 controls bitten or infested by normal uninfected lice, have all remained healthy. In the great majority of the experiments the lice were pure bred and were hatched from ova brought from England.

We have previously reported to you the method of infection, but it may be advisable to repeat here that three groups of lice numbering from 25 to 100 adult lice each, have been placed on the trench fever cases as follows :

Group 1, feeding during the first and second days of the fever,
2, feeding from the 3rd to 6th days of the fever,
3, feeding for the first week of the disease.

These three groups as they were removed from the patient with trench fever were placed upon a volunteer. In one series of experiments they were all to remain upon the volunteer until he contracted trench fever or for a period of 30 or more days. In a second series of experiments one-half of them were to be removed from the volunteer after from 48 to 60 hours from the time they had bitten the original case of trench fever, and were then to be placed upon a second volunteer to remain until he developed trench fever, or, if he did not develop trench fever, for 30 days. These two series of experiments have been performed in order to exclude purely direct mechanical transmission of the blood and organism by the louse.

In another series of experiments planned, the interval between the biting of the infected patient and second volunteer has been extended to six days[1].

In still another series of experiments comprising three volunteers, so-called " wild lice " were employed for infection; that is, lice taken from the clothing of trench fever cases. A fourth series of experiments is being performed to see if there is hereditary transmission of the virus of trench fever in the louse.

In the majority of the experiments the lice have been placed in a covered cell made of calico, strapped upon the arm with adhesive plaster. Into this cell is placed a small piece of flannel shirt. The cell measures inside from 3 to 4 inches long by about 2 inches wide. In it the lice live naturally, clinging to the fibrous cloth, and at other times wandering to the skin, feeding and depositing their excrement. They also breed and lay ova, some of the latter of which hatch in the cell. At intervals the cell was removed, the lice examined, and the patient, in some instances, was allowed to scratch the area if he so desired. In this way perhaps louse excrement sometimes entered slight abrasions caused by the scratching. Often a crushed louse was found in the cell, for the patient might scratch himself with the cell on his arm. In other words, if the individual contracted the disease in this manner from the louse it was evidently by a natural mode of infection. So far 12 of the individuals who have harbored the infected lice in this manner have contracted the disease. The time which elapsed from the date when the lice were first placed upon these individuals to the date of the beginning of the disease has been : 1 case 16 days; 1 case 17 days; 2 cases 19 days; 1 cases 20 days; 2 cases 21 days; 1 case 25 days; 3 cases 26 days[2].

The period from the time the lice were put upon the patient to the date of the development of the disease, as would be expected, is somewhat longer than the incubation period in the majority of the cases in which the disease was produced by the injection of a large dose (10 cc.) of the blood, though in two instances, as noted, the individual inoculated with whole blood did not develop the disease until about the 20th day after inoculation.

The disease produced by inoculation of the blood or by the louse infection is apparently the same. In the four men, Nos. 23, 9, 28, and 52, who have developed the disease, it should be noted that the lice employed in infecting them were allowed to feed first upon

1. It was originally planned in case the louse was found to convey the infection to test its infectivity at intervals of 48 hours up to the limit of time of such infectivity.

2. Since this report was read transmission of the infection by the louse has been demonstrated in 2 more cases with incubation periods of 30 and 35 days respectively.

four trench fever cases. They were then after an interval placed upon four different volunteers, Nos. 5, 39, 47, and 13, for approximately 2 days. One half of them were then removed from these men and placed upon Nos. 23, 9, 28, and 52, who developed the disease after 19, 26, 20, and 20 days respectively. Apparently, therefore, the transmission of the disease by the louse is not necessarily direct and mechanical.

The cases of experimentally produced trench fever have been seen, and the diagnosis of the disease confirmed, not only by ourselves but also by the members of the Trench Fever Commission of the B. E. F., consisting of Sir William Leishman, Chairman of the Commission, Major General Sir Wilmot Herringham, Major General Sir John Rose Bradford, and Colonel W. O. Beveridge, as well as by a number of other prominent clinicians.

Clinical laboratory serological and bacteriological examinations of blood, and of faeces, and urine of each patient employed and of each volunteer who has developed the disease, have also been made. The investigations and experiments have also demonstrated that trench fever is a specific disease and is not a form of enteric fever.

They have also confirmed certain clinical knowledge of the disease. These and other points will be discussed fully in a complete report.

To summarize, it has been shown that the organism causing Trench Fever is present in the plasma of the blood and up to the present time it has not been shown to be filterable (though in regard to the filterability of the virus, sufficient time has not elapsed in the inoculated subjects for us to give a final decision). The disease is transmitted naturally by the louse *Pediculus corporis*, and apparently this is the important and common means of transmission. The experiments are still in progress and a report will be made later of further results.

We wish to express our thanks and appreciation to our British colleagues for their cooperation and assistance in this work and particularly to General Sir William Macpherson, Assistant Director General Medical Services, B. E. F., to Sir William Leishman, General Sir Wilmot Herringham, and the other members of the Medical Investigation Committee, B. E. F., and also to Colonel B. Burke, Commanding Officer, Stationary Hospital No..., B. E. F., to Major H. Drummond, Captain R. Urwick, and Captain W. Perkins, R. A. M. C., and the other members of the staff of this hospital who have cooperated with us; also to Mr. A. W. Bacot, of the Lister Institute, for providing on two occasions lice and eggs from his pure stock.

We are am also indebted to the authorities of the Service de Santé of the French Army, for the use of a mobile bacteriological laboratory, of the Garstun-Tilmant type.

We wish also to thank General A. E. Bradley, Chief Surgeon, American E. F., Colonel M. W. Ireland, U. S. A., M. C., and Major Alexander Lambert, Chief Surgeon, American Red Cross, for having made these investigations possible. I cannot speak too highly of the work of the medical officers who have been engaged in these researches with me, Major H. F. Swift, Captain E. L. Opie, Captain Walter Baetjer, Captain Ward J. MacNeal, Captain Alvin M. Pappenheimer, M. R. C., and Lieutenant Alexander Peacock, R. A. M. C. I can only add that they have rendered invaluable assistance in these researches.

I wish also to express my appreciation of the services of Lieutenant David Rapport, N. A., for assistance in the clinical work, and of those of Lieutenant E. Meadows of the American Red Cross, which he has rendered as secretary of the commission.

Words fail me in attempting to express admiration of the morale and courage of the volunteers. They have more than done their part in addition by their evident desire to cooperate in every way possible towards making the experiments accurate, and I trust that some recognition will be made of the sacrifices they have endured in order to save man power foı the army, and to relieve the suffering of their fellow men.

